# Efficacy and Safety of Gel Immersion Endoscopic Mucosal Resection for Gastric Neoplasms in Patients With Familial Adenomatous Polyposis: A Multicenter Retrospective Study

**DOI:** 10.1002/deo2.70209

**Published:** 2025-09-24

**Authors:** Hidenori Kimura, Kohei Shigeta, Yohei Yabuuchi, Yoichi Yamamoto, Soichiro Nagao, Akito Noguchi, Shinya Uematsu, Shuhei Shintani, Hiroto Inoue, Atsushi Nishida, Hiroyuki Ono, Osamu Inatomi

**Affiliations:** ^1^ Department of Medicine Division of Digestive Endoscopy Shiga University of Medical Science Otsu Japan; ^2^ Division of Endoscopy Shizuoka Cancer Center Nagaizumi Japan; ^3^ Department of Gastroenterology Kobe City Medical Center General Hospital Kobe Japan; ^4^ Department of Medicine Division of Gastroenterology Shiga University of Medical Science Otsu Japan

**Keywords:** endoscopic submucosal dissection, familial adenomatous polyposis, gastric neoplasms, gel immersion endoscopic mucosal resection, treatment outcomes

## Abstract

**Objectives:**

Approximately 10%–30% patients with familial adenomatous polyposis (FAP) develop gastric neoplasms (GNs). Although recent reports have suggested the effectiveness of gel‐immersion endoscopic mucosal resection (GI‐EMR) for FAP‐associated GNs, given its simplicity and safety, treatment outcomes for such lesions have not been evaluated. We aimed to investigate the efficacy and safety of GI‐EMR compared with endoscopic submucosal dissection (ESD) for GNs in patients with FAP.

**Methods:**

In this multicenter, retrospective study, treatment outcomes of ESD and GI‐EMR for GNs measuring ≤20 mm with protruding or flat elevated morphology between April 2011 and November 2024 were compared.

**Results:**

This study included 15 ESD and 12 GI‐EMR cases. En bloc and R0 resection rates did not significantly differ between the ESD and GI‐EMR groups (100% vs. 100% and 100% vs. 83.3%, *p* = 0.19, respectively). The procedure time was significantly shorter for GI‐EMR than for ESD (2 vs. 47 min, *p* < 0.001, respectively). Intraprocedural perforation occurred in 6.7% of ESD cases, but was not observed in the GI‐EMR group. Neither delayed bleeding nor perforation occurred in any group. During the median follow‐up period of 22.3 months, no local recurrence was observed in either group.

**Conclusions:**

GI‐EMR may be considered one of the therapeutic options for GNs in patients with FAP.

## 1 Introduction

Familial adenomatous polyposis (FAP) is a hereditary disorder caused by pathogenic germline mutations in the adenomatous polyposis coli (APC) gene, with an estimated prevalence ranging from one in 8500 to 20,000 individuals [1, 2, 3]. FAP not only causes neoplasms in the colon but also affects other parts of the gastrointestinal tract, with a reported gastric neoplasm (GN) incidence of 10%–30% [4, 5, 6, 7, 8, 9]. Although recent studies have reported the efficacy of intensive endoscopic therapeutic interventions for colorectal and duodenal neoplasms in FAP [10, 11], reports on such interventions for GNs remain limited [2, 7, 12, 13]. Furthermore, there are currently no established guidelines for endoscopic resection of GNs associated with FAP, and the optimal resection method has not been determined.

Recently, the effectiveness of gel immersion endoscopic mucosal resection (GI‐EMR) using Viscoclear (Otsuka Pharmaceutical Factory, Tokushima, Japan), a gel designed for securing the endoscopic field of view, has been reported [14, 15, 16]. This method maintains the simplicity of underwater EMR (UEMR) using a gel instead of water, allowing for a clear field of view even in the presence of residue or mucus, enabling snaring under direct visualization. A previous report demonstrated that GI‐EMR in the duodenum achieved significantly shorter procedure times and higher R0 resection rates compared with UEMR [17]; it is expected to improve treatment outcomes for GNs as well. A previous report suggested the effectiveness of GI‐EMR for GNs associated with FAP [14], indicating its potential as an endoscopic therapeutic intervention for such lesions. However, no previous studies have evaluated treatment outcomes of GI‐EMR for FAP‐associated GNs.

This study aimed to investigate the efficacy and safety of GI‐EMR for GNs in patients with FAP compared with conventional resection methods.

## 2 Methods

### 2.1 Study Design and Patients

In this multicenter retrospective study, we extracted records of patients with FAP who underwent endoscopic resection for GNs between April 2011 and November 2024 at three institutions (Shiga University of Medical Science Hospital, Shizuoka Cancer Center, and Kobe City Medical Center General Hospital). The following diagnostic criteria for FAP were established: presence of ≥100 adenomatous polyps in the colon and rectum, presence of 10–99 adenomas accompanied by a family history of FAP, or identification of a pathogenic germline mutation in the APC gene [3]. Moreover, our cohort included some cases from a previous case series study [16].

Until 2022, endoscopic submucosal dissection (ESD) was mainly performed for GNs in patients with FAP at our institutions, as conventional EMR was reported to have a higher rate of piecemeal resection and recurrence compared with ESD, even for GNs measuring ≤20 mm [18]. Conventional EMR was indicated only for pedunculated lesions. Since 2022, following reports on the efficacy of gastric GI‐EMR [14, 19], the method has been mainly performed for elevated GNs measuring ≤20 mm, located at or near the greater curvature of the upper or middle third of the stomach [16]. UEMR is generally not performed at our three institutions because of concerns regarding poor visibility owing to water mixing with gastric mucus or residue over time [16]. Treatment decisions were finalized following discussions at a gastroenterology conference. Before the procedure, all patients provided written informed consent for endoscopic treatment.

Although there is no clearly defined indication for FAP‐associated GNs, endoscopic treatment was mainly performed for lesions suspected to be intramucosal cancers or adenomas measuring ≥10 mm. Although there is currently no clear consensus regarding the optimal resection method for FAP‐associated GNs [13], ESD, which has been widely performed for sporadic GNs (even those measuring ≤20 mm), was provisionally adopted as the standard treatment for FAP‐associated GNs in this study. Based on this provisional standard, we conducted a comparative analysis with the novel technique of GI‐EMR. To assess the treatment outcomes of GI‐EMR and ESD, we extracted cases of GNs measuring ≤20 mm in diameter, with protruding or flat elevated morphologies, which were indications for GI‐EMR at our institution, from patients undergoing endoscopic resection during the study period. Treatment outcomes for lesions at the greater curvature, which were considered the most appropriate indications for GI‐EMR, were also evaluated.

This study was approved by the Institutional Review Board of Shiga University of Medical Science (Institutional number: R2024‐101). Informed consent was obtained using the opt‐out method.

### 2.2 Endoscopic Procedures and Patient Management

For all procedures, we used an endoscope (GIF‐H290T, GIF‐Q260J, GIF‐H290Z, GIF‐H260Z, or GIF‐2TQ260M [Olympus Medical Systems]) with a distal attachment (D201–11804 [Olympus Medical Systems] or elastic touch [Top]) and a standard electrosurgical generator (VIO300D or VIO3 [ERBE]). Intravenous sedation and analgesia without endotracheal intubation were performed in each treatment.

**FIGURE 1:**
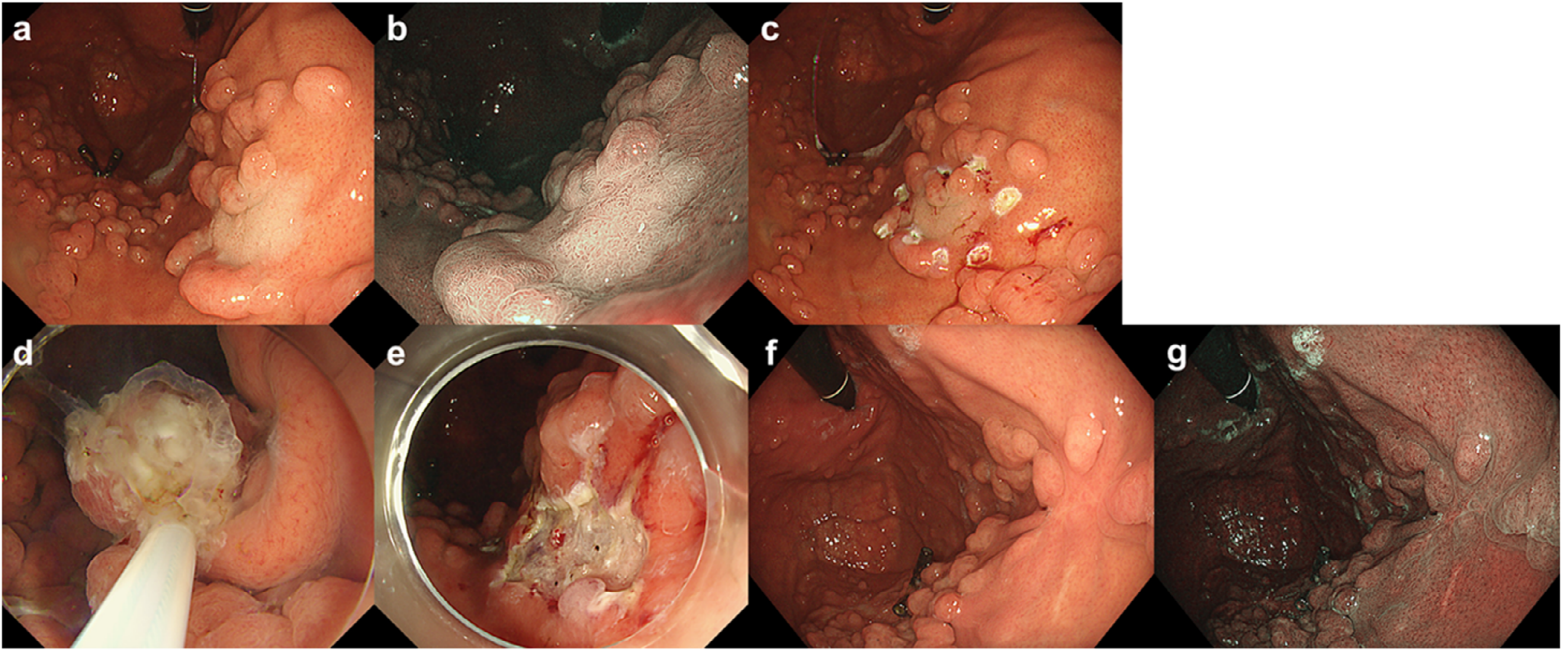
GI‐EMR procedure. (a) A whitish flat elevated lesion at the anterior wall of the upper gastric body with fundic gland polyposis in a patient with FAP. (b) NBI view. (c) Marking was performed. (d) Snaring under gel immersion. (e) Mucosal defect without perforation. (f, g) No residual tumor at the post‐EMR ulcer scar in follow‐up endoscopy. GI‐EMR, gel immersion endoscopic mucosal resection; FAP, familial adenomatous polyposis; NBI, narrowband imaging.

Detailed procedure for ESD has previously been described [20, 21]. Briefly, a solution containing 0.4% sodium hyaluronic acid (Muco Up; Boston Scientific Japan, Tokyo, Japan) mixed with saline and indigo carmine was injected in the submucosal layer. After marking around the lesion, a mucosal incision and submucosal dissection were performed using a needle‐type knife or an insulated‐tip knife in combination with Endocut, swift coagulation, or forced coagulation modes. For GI‐EMR, we measured the lesion size using forceps or snares with predetermined diameters and marked the area around the lesion using a snare tip or needle knife before resection. A 50 mL syringe was connected to a BioShield irrigator (U.S. Endoscopy) to deliver the gel (ViscoClear [Otsuka Pharmaceutical Factory, Tokushima, Japan]) through the accessory channel of the endoscope into the stomach. The lesion was grabbed with a snare and excised using a blended‐cut current (Endocut Q; effect 3, time interval 2, time duration 2) (**Figure** **1**). Depending on lesion size and characteristics, either a 10 or 15 mm oval snare (SnareMaster Plus [Olympus Medical Systems]) or a 15 or 20 mm rounded stiff snare (Captivator II [Boston Scientific]) was used. We did not routinely use an over tube. Following resection, specimens were preserved in 10% formalin and sent to the Pathology Division for histological examination. Both ESD and GI‐EMR were available at all the participating institutions.

After endoscopic resection, patients were monitored during their hospital stay for 3–5 days. In cases where R0 resection was confirmed, follow‐up endoscopy was scheduled between 6 months and 1 year after the procedure. If the pathological evaluation revealed positive or unclear margins, follow‐up endoscopy was performed within 6 months to obtain biopsies from the ulcer scar at the resection site.

### 2.3 Data Collection and Definition

Clinicopathological data were retrospectively extracted from the institutional electronic medical records. En bloc resection rate, R0 resection rate, procedure time, and adverse events, including delayed bleeding and perforation, were evaluated.

En bloc resection was defined as the complete removal of the lesion in a single piece. R0 resection was defined as en bloc resection with histologically tumor‐free margins. Procedure time was measured from the initiation of irrigating gel application to the completion of lesion removal for GI‐EMR, and from the initiation of submucosal injection to the completion of lesion resection for ESD. Intraprocedural bleeding was defined as bleeding during endoscopic resection requiring endoscopic or surgical hemostasis. Delayed bleeding was defined as hematemesis or melena occurring after endoscopic resection necessitating blood transfusion or additional therapeutic intervention. Perforation was defined as a visible full‐thickness defect following endoscopic resection. Local recurrence was defined as a recurrent neoplasm at the ulcer scar after endoscopic resection, confirmed by histological examination.

### 2.4 Statistical Analysis

Continuous variables are presented as medians with interquartile ranges (IQRs). Categorical variables are presented as numbers and percentages. Continuous variables were compared using the Mann–Whitney U test, whereas Fisher's exact test was used for categorical variables. Statistical significance was set at *p*‐value <0.05. All statistical analyses were conducted using EZR software version 1.64 (Saitama Medical Center, Jichi Medical University, Saitama, Japan).

## 3 Results

### 3.1 Baseline Characteristics

**FIGURE 2:**
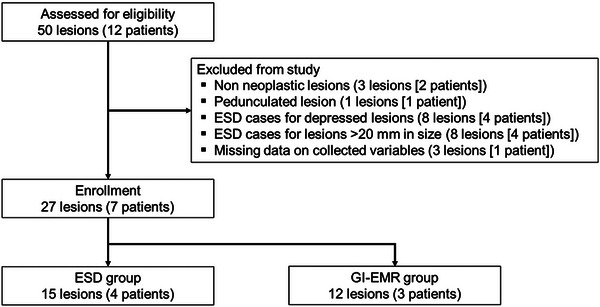
Flow chart of patient selection. ESD, endoscopic submucosal dissection; GI‐EMR, gel immersion endoscopic mucosal resection.

The patient selection flowchart is shown in **Figure** **2**. Overall, 27 GNs (seven patients), sized ≤20 mm with flat elevated, or protruding morphology, in which ESD or GI‐EMR was performed, were included, consisting of 15 ESD and 12 GI‐EMR cases. Patient baseline characteristics are summarized in **Table** **1**. The majority of lesions in both groups were located in the upper or middle third of the stomach. The lesion size did not significantly differ between groups (10 mm vs. 11.5 mm, respectively; *p* = 0.30).

**TABLE 1:** Baseline characteristics.

	ESD	GI‐EMR	
**Patients**	***N* = 4**	***N* = 3**	***p*‐Value**
Age, years, median (IQR)	52 (44–61)	42 (33–47)	0.212
Men (%)	1 (25)	0 (0)	1.00
Helicobacter pylori status			1.00
Uninfected	3 (75)	3 (100)	
Infected	0 (0)	0 (0)	
Eradicated	1 (25)	0 (0)	
Atrophic gastritis			1.00
None	3 (75)	3 (100)	
Closed type ^*^	1 (25)	0 (0)	
Open type	0 (0)	0 (0)	

	**ESD**	**GI‐EMR**	
**Lesions**	***N* = 15**	***N* = 12**	***p*‐Value**
Lesion size, mm, median (IQR)	10 (5–17.5)	11.5 (10–13.5)	0.300
Location 1 (%)			0.037
Upper third	7 (47)	11 (92)	
Middle third	7 (47)	1 (8.3)	
Lower third	1 (6.7)	0 (0)	
Location 2 (%)			0.036
Greater curvature	4 (27)	9 (75)	
Lesser curvature	4 (27)	0 (0)	
Anterior wall	6 (40)	2 (17)	
Posterior wall	1 (6.7)	1 (8.3)	
Morphology (%)			0.087
Protruding	2 (13)	6 (50)	
Flat elevated	13 (87)	6 (50)	
Antithrombotic agents (%)	0 (0)	0 (0)	N/A
Certified endoscopists performed	15 (100)	10 (83)	0.188
Pathology of biopsy specimen before treatment (%)			0.022
Adenocarcinoma	8 (53)	1 (8.3)	
Adenoma	6 (40)	10 (83)	
Non‐neoplastic	1 (6.7)	0 (0)	
Biopsies were not performed	0 (0)	1 (8.3)	

Abbreviations: ESD, endoscopic submucosal dissection; GI‐EMR, gel immersion endoscopic mucosal resection; IQR, interquartile range; N/A, not available.

* One patient with closed‐type atrophic gastritis underwent subtotal stomach‐preserving pancreaticoduodenectomy for ampullary carcinoma, and therefore had a remnant stomach thereafter.

### 3.2 Treatment Outcomes and Adverse Events

Treatment outcomes of ESD and GI‐EMR are presented in **Table** **2**. En bloc and R0 resection rates did not significantly differ between groups (100% vs. 100% and 100% vs. 83.3%, *p* = 0.19, respectively). The procedure time was significantly shorter for GI‐EMR than for ESD (2 min vs. 47 min, respectively; *p* < 0.001). GI‐EMR was completed using 200 g of gel (one pack of ViscoClear) in all cases. Even when the analysis was limited to lesions on the greater curvature (**Table** **3**), accounting for 75% of the GI‐EMR cases, all lesions in both groups were located in the upper third of the stomach, and treatment outcomes showed a trend similar to that described above.

**TABLE 2:** Treatment outcomes.

	ESD	GI‐EMR	
	***N* = 15**	***N* = 12**	***p*‐Value**
En bloc resection (%)	15 (100)	12 (100)	N/A
R0 resection (%)	15 (100)	10 (83)	0.188
Horizontal margin (%)			0.188
HM0	15 (100)	10 (83)	
HMX	0 (0)	2 (17)	
HM1	0 (0)	0 (0)	
Vertical margin (%)			N/A
VM0	15 (100)	12 (100)	
VMX	0 (0)	0 (0)	
VM1	0 (0)	0 (0)	
Procedure time, min, median (IQR)	47 (32–60.5)	2.0 (2.0–3.3)	<0.001
Pathology (%)			<0.001
Intramucosal cancer	12 (80)	1 (8.3)	
Adenoma	3 (20)	11 (92)	
Adverse events			
Intraprocedural bleeding (%)	N/A	2 (17)	N/A
Intraprocedural perforation (%)	1 (6.7)	0 (0)	1.00
Delayed bleeding (%)	0 (0)	0 (0)	N/A
Delayed perforation (%)	0 (0)	0 (0)	N/A
Local recurrence (%) ^*^	0 (0)	0 (0)	N/A

Abbreviations: ESD, endoscopic submucosal dissection; GI‐EMR, gel immersion endoscopic mucosal resection; IQR, interquartile range; N/A, not available.

* Median follow‐up period: 22.3 months (IQR: 14.5–78.3 months)

**TABLE 3:** Treatment outcomes for gastric neoplasms located at the greater curvature.

	ESD	GI‐EMR	
	***N* = 4**	***N* = 9**	***p*‐Value**
Characteristics			
Lesion size, mm, median (IQR)	17.5 (13.8–20)	11 (10–13)	0.156
Location 1 (%)			N/A
Upper third	4 (100)	9 (100)	
Middle third	0 (0)	0 (0)	
Lower third	0 (0)	0 (0)	
Morphology (%)			0.105
Protruding	0 (0)	5 (56)	
Flat elevated	4 (100)	4 (44)	
Certified endoscopists performed	4 (100)	7 (78)	1.00
Outcomes			
En bloc resection (%)	4 (100)	9 (100)	N/A
R0 resection (%)	4 (100)	7 (78)	1.00
Procedure time, min, median (IQR)	60.5 (54.3–63.5)	2.0 (2.0–3.0)	0.005
Pathology (%)			0.014
Intramucosal cancer	3 (75)	0 (0)	
Adenoma	1 (25)	9 (100)	
Adverse events			
Intraprocedural bleeding (%)	N/A	2 (22)	N/A
Intraprocedural perforation (%)	0 (0)	0 (0)	N/A
Delayed bleeding (%)	0 (0)	0 (0)	N/A
Delayed perforation (%)	0 (0)	0 (0)	N/A
Local recurrence (%)	0 (0)	0 (0)	N/A

Abbreviations: ESD, endoscopic submucosal dissection; GI‐EMR, gel immersion endoscopic mucosal resection; IQR, interquartile range; N/A, not available.

Regarding adverse events, intraprocedural perforation occurred in 6.7% of patients undergoing ESD; however, no such cases were observed in the GI‐EMR group. Intraprocedural bleeding occurred in two GI‐EMR cases, which could be managed endoscopically. Neither delayed bleeding nor perforation occurred in any group.

The two lesions in which R0 resection was not achieved exhibited unclear horizontal margins; however, the vertical margins were negative. During the median follow‐up period of 22.3 months (IQR: 14.5–78.3 months), no local recurrence was observed in either group.

## 4 Discussion

To the best of our knowledge, this is the first study evaluating the treatment outcomes of GI‐EMR compared with conventional resection methods for GNs in patients with FAP. This study demonstrated that GI‐EMR resulted in favorable treatment outcomes, including en bloc resection and no local recurrence, with a shorter procedure time compared with ESD. Regarding adverse events, intraprocedural perforation was observed in the ESD group but not in the GI‐EMR group. Neither delayed bleeding nor perforation occurred in any group. Hence, GI‐EMR may be a safe and effective therapeutic intervention for GNs in patients with FAP.

The abovementioned findings suggest that GI‐EMR may be a viable alternative with an efficacy similar to that of ESD, but with fewer limitations. Although ESD has been widely performed for GNs because of its higher en bloc and R0 resection rates and lower local recurrence rate compared with conventional EMR [18], it requires advanced technical skills and a longer procedure time with a higher risk of adverse events such as perforation and bleeding. In contrast, EMR has long been performed as a simple and effective treatment [22]. Furthermore, UEMR (first reported in 2012) [23] has shown lower rates of piecemeal resection and local recurrence compared with conventional EMR in the colon [24, 25]. Its usefulness and simplicity of not requiring submucosal injections led to its rapid adoption worldwide [26]. Although the usefulness of UEMR for the stomach has also been reported [27], it has some limitations: poor visibility owing to mixture with mucus, and backflow into the esophagus and pharynx due to prolonged water immersion, causing aspiration. Therefore, we focused on GI‐EMR [14], using gel instead of water, allowing for a clear field of view without mixing with residue. In addition, even in the event of immediate post‐resection bleeding, gel helps maintain good visualization, facilitates confirmation of any residual lesion at the ulcer margin, and enables prompt hemostasis. These advantages may have contributed to better treatment outcomes while retaining the simplicity of UEMR. In fact, a previous study evaluating the clinical outcomes of GI‐EMR [16] demonstrated similar R0 resection rates for gastric adenocarcinomas and adenomas, with a trend toward shorter procedure time compared with previously reported results for gastric UEMR [27]. Furthermore, although aspiration pneumonia after gastric UEMR has been reported in 3.1% of cases (one out of 32 lesions) [27], no cases of aspiration pneumonia in gastric GI‐EMR have been reported [16] under conditions where over tubes are not used in routine practice. Moreover, the treatment outcomes of GI‐EMR for GNs measuring ≤20 mm in diameter, with protruding or flat elevated morphology in patients with FAP, were similar to those of conventional EMR and UEMR (**Table** **4**) [13]. Consequently, GI‐EMR appears to be a preferable approach among various endoscopic resection techniques for managing elevated gastric lesions measuring ≤20 mm.

**TABLE 4:** Treatment outcomes of endoscopic resection for gastric neoplasms measuring ≤20 mm in diameter with protruding or flat elevated morphology in patients with familial adenomatous polyposis: a comparison between the present study and a previous report.

	Shimamoto et al. [13]		Present study	
Lesion size (mean or median)	10 mm	12 mm	10 mm	11.5 mm
Endoscopic procedure	CEMR (*N* = 12)	UEMR (*N* = 25)	ESD (*N* = 15)	GI‐EMR (*N* = 12)
En bloc resection rate	92%	88%	100%	100%
R0 resection rate	75%	56%	100%	83%
Median procedure time, min (IQR)	5 (3–8)	2 (2–3)	47 (32–61)	2 (2–3)
Intraprocedural perforation	N/A	N/A	6.7%	0%
Delayed bleeding	N/A	N/A	0%	0%
Delayed perforation	N/A	N/A	0%	0%

Abbreviations: CEMR, conventional endoscopic mucosal resection; ESD, endoscopic submucosal dissection; GI‐EMR, gel immersion endoscopic mucosal resection; IQR, interquartile range; N/A, not available; UEMR, underwater endoscopic mucosal resection.

No severe adverse events, such as delayed bleeding or perforation, were observed during GI‐EMR, while intraprocedural perforation was observed in 6.7% of the ESD group. All lesions in the ESD group were treated by experts; hence, the complication rate was lower than in a previous report [28]. FAP‐associated GNs are often accompanied by fundic gland polyposis, posing a challenge during mucosal incisions during ESD. In fact, lesions surrounded by fundic gland polyposis are associated with a higher risk of intraprocedural perforation during ESD than those without fundic gland polyposis [28]. Moreover, FAP‐associated GNs are known to frequently have synchronous and metachronous neoplasms after resection [28]. Therefore, unlike sporadic gastric cancer, FAP‐associated GNs can develop in other parts of the stomach, even if they are completely resected by ESD. Although ESD is an effective treatment enabling precise and complete resection of FAP‐associated gastric tumors, its high complication rates and potential risks associated with repeated procedures suggest the need for a less invasive treatment approach if the local recurrence rate remains unchanged. In this study, although some cases treated with GI‐EMR showed unclear horizontal margins, no local recurrence was observed. Additionally, the procedure time was significantly shorter than that for ESD, and no severe complications were reported. Furthermore, unlike ESD, GI‐EMR does not require various devices, thereby reducing costs. These findings suggest that GI‐EMR is a simple, effective, and repeatable treatment option for patients with FAP‐associated GNs.

We considered protruding or flat elevated lesions located at or near the greater curvature with a diameter of ≤20 mm as good indications for gastric GI‐EMR in patients with FAP. First, the greater curvature is considered a challenging site for ESD because of its tendency to submerge as it is on the gravity‐dependent side; however, this location is advantageous for GI‐EMR because it facilitates easy snaring and allows water or gel to accumulate, enhancing GI‐EMR feasibility. Second, GI‐EMR is preferably performed for protruding or flat elevated lesions because a depressed morphology increases the likelihood of slippage during snaring. Moreover, GNs associated with FAP are frequently surrounded by fundic gland polyposis, posing significant challenges for mucosal incision in ESD. However, in GI‐EMR, the surrounding fundic gland polyposis is easily captured by the snare, facilitating easier lesion removal. Thus, FAP‐associated GNs may be good candidates for GI‐EMR. As for elevated sporadic GNs, since there is no surrounding fundic gland polyposis, the snare may slip more easily compared with FAP‐associated GNs. In fact, a previous study has reported favorable outcomes of GI‐EMR for sporadic GNs less than 10 mm [29]. Therefore, although we set 20 mm as the cutoff for GI‐EMR in this study, following the EMR size criteria recommended in the guidelines [22], the size criteria for GI‐EMR may need to be adjusted depending on whether the GN is associated with FAP or is sporadic.

This study has some limitations. First, the retrospective study design and small sample size may have introduced selection bias. Second, a significant difference was observed in the background factors between groups, raising concerns about the validity of the comparison. However, in contrast to the previous report [16], which was a case series of various lesions treated with GI‐EMR, this study focused on GNs in patients with FAP, which are more frequently synchronous or metachronous than usual and considered to require minimally invasive therapeutic intervention. In this study, we demonstrated the outcomes of GI‐EMR and clarified its usefulness for the first time. Therefore, our study may help in understanding the potential role of GI‐EMR for such lesions. Moreover, by presenting the treatment outcomes of the conventional resection method, ESD, as a reference, our results may also have clinical implications for endoscopists who hesitate to perform EMR due to concerns about piecemeal resection or local recurrence of GNs, even when small, and therefore perform ESD in almost all cases. Although careful interpretation is required, we believe the present study provides preliminary but meaningful data that justifies further validation through a prospective multicenter trial. Third, ESD was mainly performed for FAP‐associated GNs at the participating institutions until GI‐EMR was introduced for GNs. Consequently, EMR and UEMR were rarely performed, and comparisons with methods other than GI‐EMR and ESD could not be performed. Hence, future studies comparing GI‐EMR to resection methods other than ESD, such as conventional EMR and UEMR, are warranted.

In conclusion, GI‐EMR may be a safe and effective therapeutic intervention for GNs in patients with FAP. Although GI‐EMR may be less invasive than ESD, the limited number of reported cases necessitates further studies to validate its safety and efficacy.

## Conflicts of Interest

The authors declare no conflicts of interest.

## Ethics Statement

**Approval of the research protocol by an Institutional Review Board**: This study was approved by the Institutional Review Board of Shiga University of Medical Science (Institutional number: R2024‐101).

## Consent

Informed consent was obtained using the opt‐out method.

## Clinical Trial Registration

N/A
